# Comprehensive surveillance data suggest a prominent role of parvovirus B19 infection in Belarus and the presence of a third subtype within subgenotype 1a

**DOI:** 10.1038/s41598-020-79587-2

**Published:** 2021-01-13

**Authors:** Marina A. Yermalovich, Alina M. Dronina, Galina V. Semeiko, Elena O. Samoilovich, Vladislav V. Khrustalev, Aurelie Sausy, Judith M. Hübschen

**Affiliations:** 1 Republican Research and Practical Center for Epidemiology and Microbiology, Minsk, Belarus; 2 Department of General Chemistry, Belarusian State Medical University, Minsk, Belarus; 3 Department of Infection and Immunity, Luxembourg Institute of Health, Esch-sur-Alzette, Luxembourg

**Keywords:** Infectious diseases, Epidemiology, Viral epidemiology

## Abstract

Human parvovirus B19 (B19V) infection is not notifiable in Belarus and its most common clinical presentation erythema infectiosum (EI) is often difficult to distinguish from other exanthematous diseases. The objective of this study was to provide comprehensive data about EI epidemiology in Belarus based on the serological and molecular investigation of samples from measles and rubella discarded cases collected between 2005 and 2019. Overall, 4919 sera were investigated for IgM antibodies against B19V and the positive cases were analysed according to year, season and age. B19V DNA was amplified by PCR in a total of 238 sera from all over the country, and sequenced for phylogenetic analyses. B19V infection was confirmed in 1377 (27.8%) measles and rubella discarded cases. Two high incidence periods and a seasonal increase of EI between mid-February to mid-July were identified. Children from 4 to 6 and from 7 to 10 years of age represented the largest groups of patients (22.51% and 22.66% of all cases, respectively), followed by adults between 20 and 29 years of age (14.23%). Among the 238 B19Vs sequenced, one belonged to subgenotype 3b and 237 to subgenotype 1a with 81 (34.2%) clustering with subtypes 1a1 and 153 (64.6%) with 1a2. Three strains (1.2%) formed an additional, well-supported cluster suggesting the presence of another subtype of 1a, tentatively named 1a3. The epidemiological and molecular analyses highlighted not only the prominent role of B19V in exanthematous diseases in Belarus, but also suggested a previously underestimated diversity of subgenotype 1a sequences with a third subtype 1a3.

## Introduction

The main clinical presentation of human parvovirus B19 (B19V) infection is erythema infectiosum (EI) or “fifth disease”, which is common in childhood but may also be observed in adults^1^. The similarity of EI to other exanthematous diseases makes its clinical diagnosis difficult and requires laboratory confirmation^2^. The importance of laboratory diagnosis of EI increased after implementation of measles and rubella elimination programs, since these infections, especially rubella, can be suspected in case of EI^3^. Therefore, laboratory verification of B19V infection has been introduced in many countries^4–8^.

In Belarus, vaccination against measles and rubella led to a significant reduction in measles incidence and to elimination of rubella and prevention of congenital rubella syndrome cases^9^. Laboratory diagnosis of B19V infection was started in Belarus in 2005 at the National laboratory as an additional investigation of samples from suspected measles and rubella cases and continues to be carried out only in this laboratory for patients from all over the country. Introduction of B19V diagnosis allowed to confirm a large outbreak in 2006^10,11^. Phylogenetic analysis of the strains collected between 2005 and 2008 showed the circulation of subgenotype 1a all over the country and allowed to discriminate two groups of sequences designated as clusters 1 and 2^10^. The worldwide existence of two genetic variants of subgenotype 1a, named subtypes 1a1 and 1a2, has since been suggested^12^ and these subtypes correspond to our clusters 2 and 1, respectively. While current evidence does not suggest any difference between subtypes concerning the clinical symptoms caused^12^, such classifications have value for epidemiological tracing and analysis^10,13^.

Here we present the results of the serological and molecular investigation of B19V infection based on samples received between 2005 and 2019, thus providing comprehensive data about EI prevalence and epidemiology in Belarus.

## Results

Of the 6318 sera collected in the frame of the nationwide measles/rubella surveillance in Belarus between 2005 and 2019, 728 (11.5%) were positive for measles IgM and 640 (10.1%) for rubella IgM (Table 1). All rubella cases and 89% of measles cases were diagnosed in persons older than 10 years of age. For the remaining 4950 cases (78.4%), both infections were excluded. In total 4919 (99.4%) serum samples were available for further testing and B19V IgM antibodies were found in 1377 (27.8%) discarded cases. B19V DNA was amplified by PCR in a total of 238 sera from all over the country, and sequenced for phylogenetic analyses.

**Table 1:** Number of samples from measles/rubella suspected patients and number and percentage of measles, rubella and B19V IgM positives by year, Belarus, 2005–2019.

Year	M/R suspected	Measles confirmed	Rubella confirmed	M/R discarded (per 100,000 population)	B19V confirmed from discarded (%)	No of genotyped B19V strains
2005	583	1	365	217* (2.2)	63 (30.6)	4
2006	754	149	229	376 (4.0)	197 (52.4)	87
2007	183	1	6	176 (1.9)	49 (27.8)	7
2008	181	0	2	179** (1.9)	16 (10.1)	8
2009	274	0	2	272 (2.9)	82 (30.1)	11
2010	191	1	0	190 (2.0)	59 (31.1)	7
2011	394	51	20	323 (3.4)	85 (26.3)	17
2012	369	10	10	349 (3.7)	84 (24.1)	19
2013	336	16	1	319 (3.4)	68 (21.3)	14
2014	437	64	1	372 (3.9)	118 (31.7)	16
2015	445	2	1	442 (4.7)	171 (38.7)	9
2016	429	10	0	419 (4.4)	181 (43.2)	12
2017	357	1	1	355 (3.8)	50 (13.9)	11
2018	758	229	2	522 (5.5)	102 (19.5)	10
2019	632	193	0	439 (4.7)	52 (11.9)	6
Total	6318	728 (11.5%)	640 (10.1%)	4950*** (3.4)	1377 (27.8)	238

*206 serum samples were available for B19V testing.

**159 serum samples were available for B19V testing.

***4919 serum samples were available for B19V testing.

### Long-term EI incidence and seasonal trends

**Figure 1:**
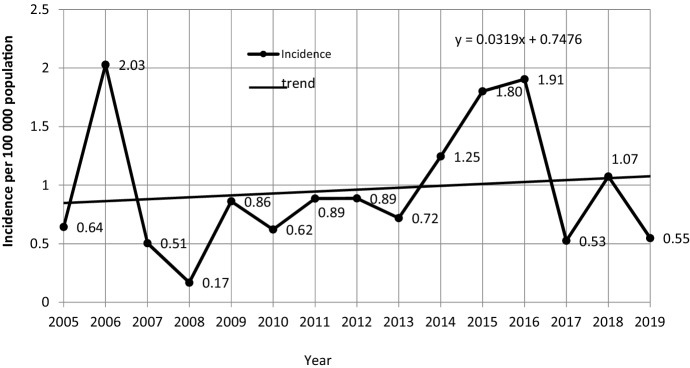
Erythema infectiosum incidence per 100,000 population in Belarus between 2005 and 2019.

The mean EI incidence during the 15-year study period was 0.96 per 100 000 inhabitants (95% CI 0.76–1.16). The lowest and highest incidence rates differed by a factor of 11.94 (0.17 in 2008 versus 2.03 in 2006) (Fig. 1).

**Figure 2:**
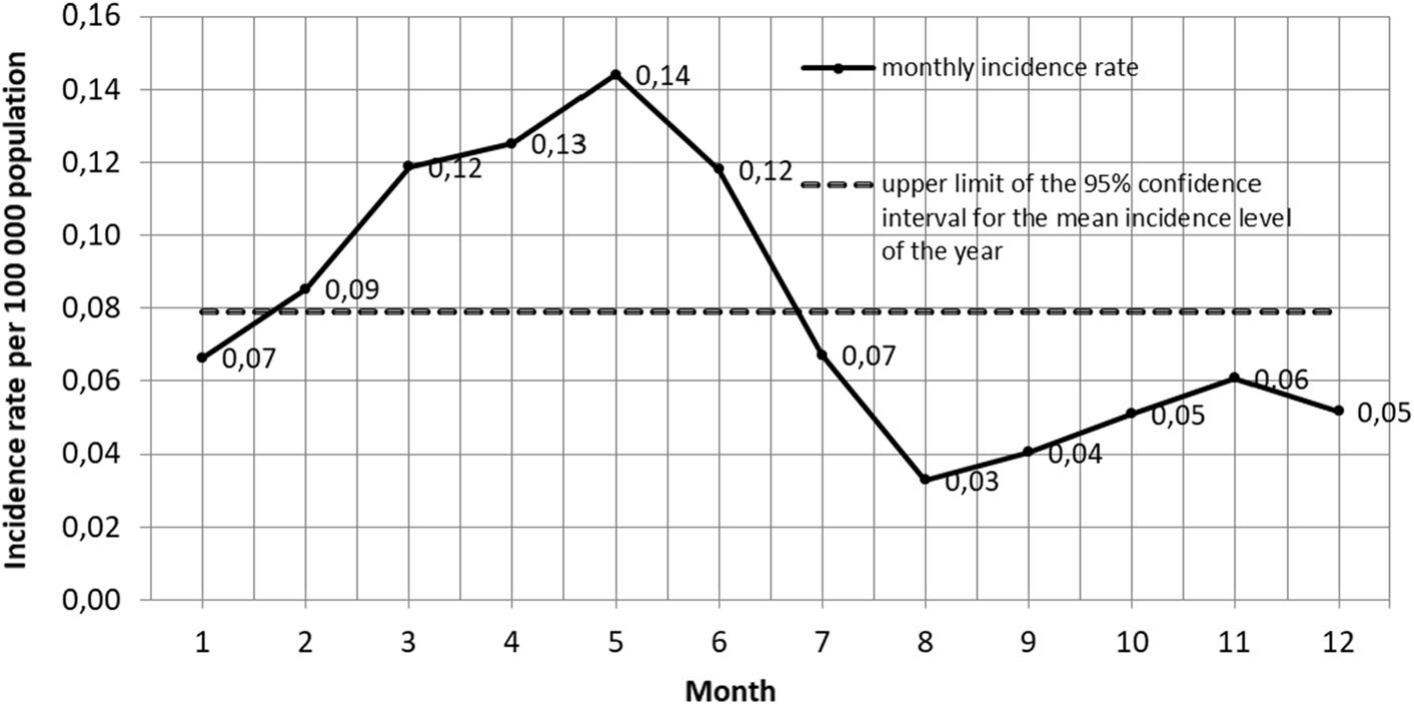
Seasonal trend of erythema infectiosum in Belarus calculated over the whole study period (2005–2019).

EI was registered throughout the year and the upper limit of the 95% confidence interval for the mean level of the year calculated over all 15 years was 0.08 (Fig. 2). The seasonal increase lasted 5 months (mid-February to mid-July), the incidence reached the maximal level of 0.14 per 100,000 inhabitants in May and the lowest rate, namely 0.03 per 100,000 inhabitants, in August. The slight increase during the autumn months did not exceed the upper limit of the annual mean (Fig. 2).

### Age distribution

**Figure 3:**
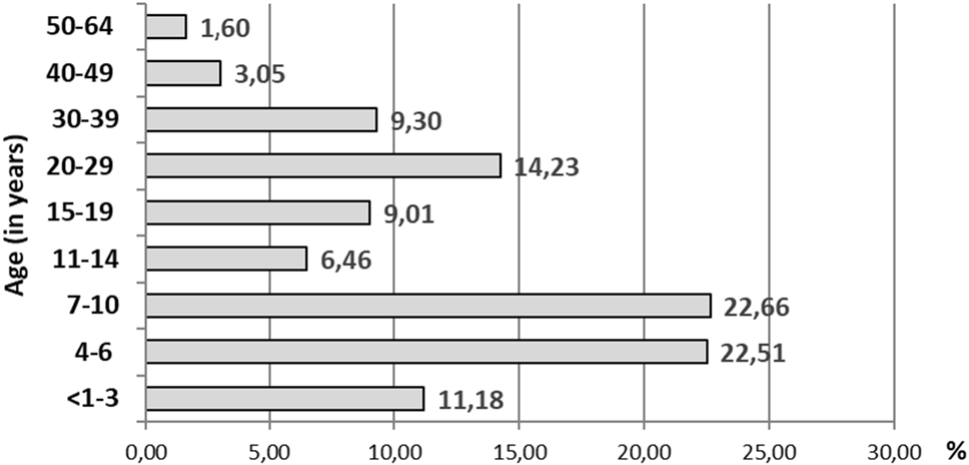
Proportion of B19V IgM positive patients by age group in Belarus between 2005 and 2019.

B19V infection was laboratory confirmed in patients from less than 1 to 64 years of age (median 13 years). In total, 62.8% (865/1377) of the patients were children up to 14 years of age, with the 4 to 6 and the 7 to 10 year-old representing the largest groups (22.51% and 22.66%, respectively). The third biggest group were adults between 20 and 29 years of age (14.23%) (Fig. 3).

**Figure 4:**
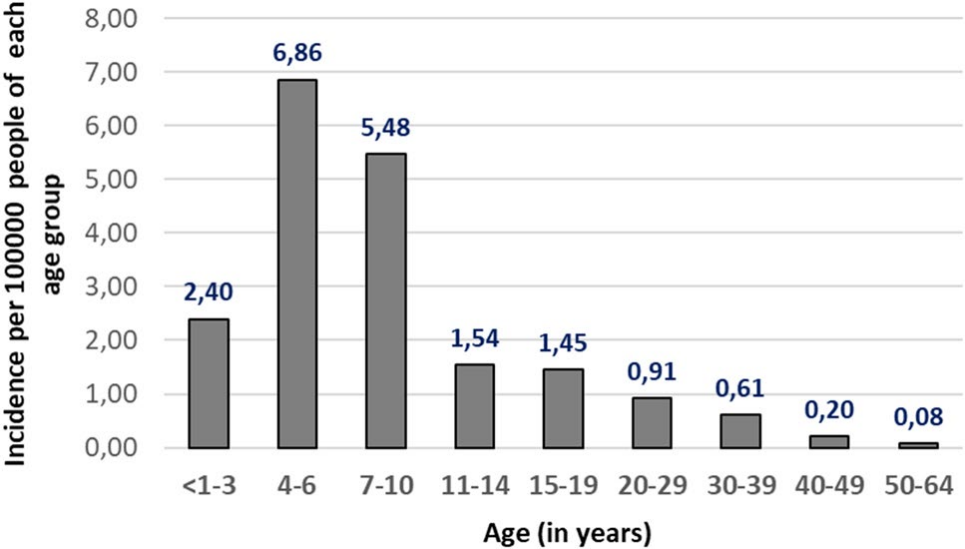
Erythema infectiosum incidence rates per 100,000 people of each age group, Belarus, 2005–2019.

The highest EI incidence rates per 100 000 people of each age group were also found in children between 4 and 6 years (6.86; 95% CI 3.90–9.82) and in 7 to 10 year-old (5.48; 95% CI 3.12–7.83) and were significantly higher (P < 0.05) than in all other age groups (Fig. 4). The incidence rates in the age groups < 1–3, 11–14, 15–19, 20–29 and 30–39 years did not differ significantly from each other (P > 0.1). The lowest rates were found in 40–49 and 50–64 year-old adults with a statistically significant difference (P < 0.01) to all other age groups (exception: there was no significant difference between the 40–49 and the 30–39 year-olds (P = 0.09)).

Based on the EI incidence rates, the patients can be divided into three groups: 0–10 year-olds (4.67; 95% CI 3.39–5.94), 11–39 year-olds (0.95; 95% CI 0.64–1.26) and 40–64 year-old (0.13; 95% CI 0.01–0.25). The risk of contracting EI was 35.8 times higher in 0–10 year-old and 7.3 times higher in 11–39 year-old compared to adults of at least 40 years of age.

### Genotype distribution

**Figure 5:**
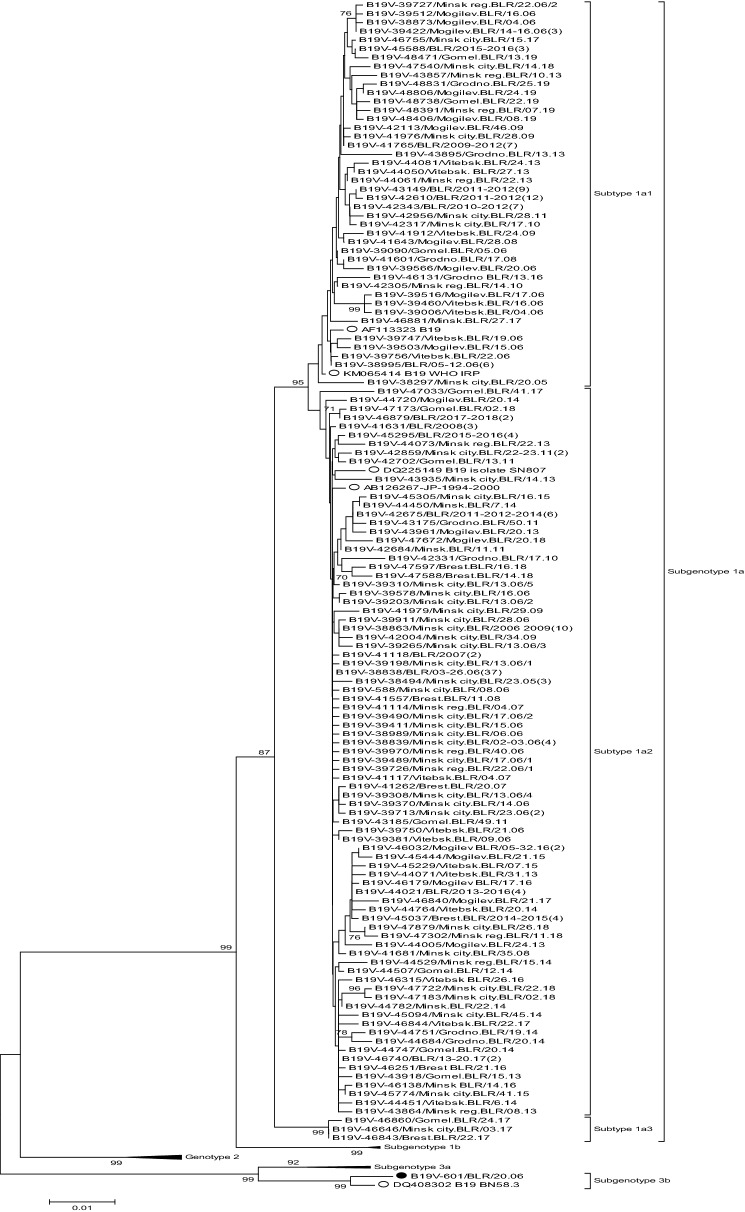
Phylogenetic tree based on 994 nt of the NS1/VP1-unique region junction including the B19V sequences from Belarus. Only one representative strain is shown for identical sequences of subtypes 1a1 and 1a2, and the total number of sequences is added behind in brackets; all three sequences of the provisional subtype 1a3 are presented; the subgenotype 3b strain is marked with a black dot. The strains are named with location, epidemiological week and year or with country name and year/years for representative strains of identical sequences isolated at different time points and/or at different locations. The reference sequences are identified by GenBank accession number and name and are marked with an open circle. Only bootstrap values ≥ 70% (1000 replicates) are shown.

Of the 238 sequences obtained between 2005 and 2019, 237 belonged to subgenotype 1a with 81 (34.2%) of them clustering with 1a1 and 153 (64.6%) with 1a2 reference sequences (Fig. 5). Three strains (1.2%) formed an additional branch suggesting a third variant of 1a. One strain, obtained in 2006 from a child with aplastic anaemia who came from Kazakhstan, belonged to genotype 3b (Fig. 5).

**Figure 6:**
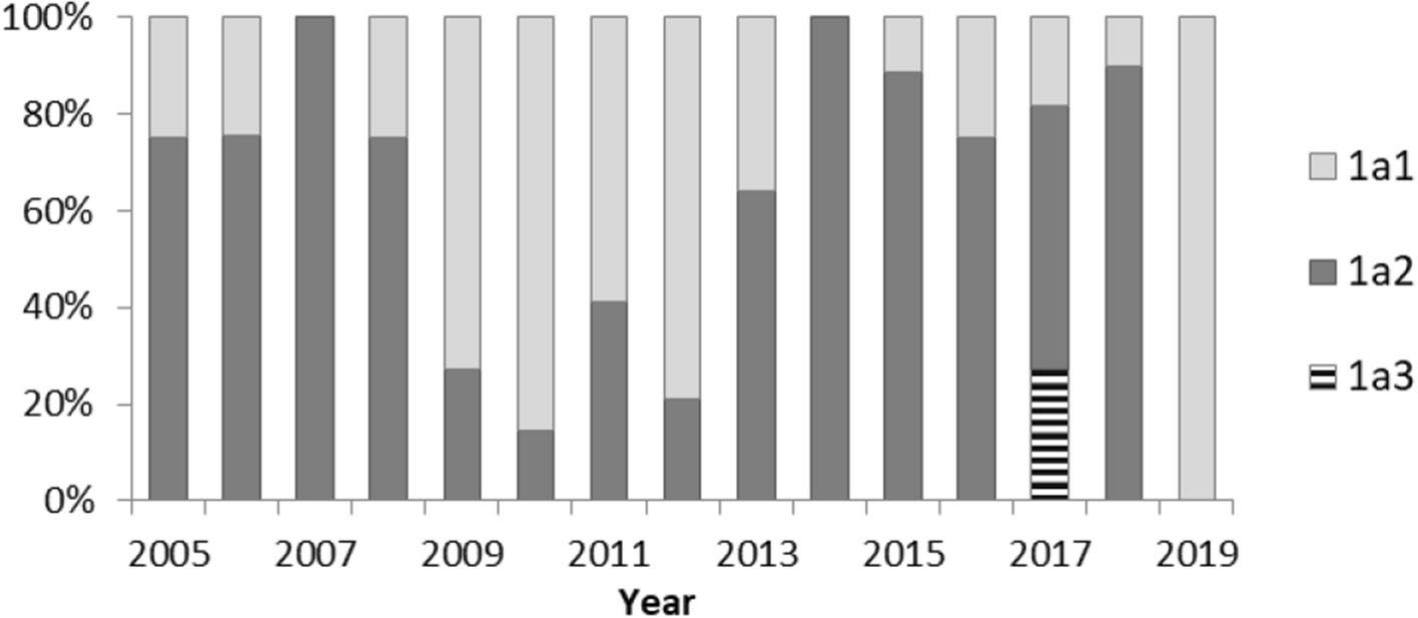
Prevalence of B19V 1a subtypes in individual years, Belarus, 2005–2019.

Both 1a1 and 1a2 subtypes circulated in Belarus with a different prevalence during individual years (Fig. 6).

At the time of the 2005/2006 epidemic, subtype 1a2 represented more than 75.0% of all strains and accounted for 100.0% and 75.0% in 2007 and 2008, when the lowest EI incidence rate was observed. Between 2009 and 2012, 1a1 dominated with 59–86% of the sequences. In 2013, the prevalence of subtype 1a2 increased again and 1a2 strains were dominant (75.0–100.0%) between 2014 and 2016, when another rise of incidence was registered, and in the two subsequent years. In the low EI incidence year 2019, 1a1 was the only subtype found.

Among the 237 sequences of 1a the mean distance was 1.02% (Table 2).

**Table 2:** Intra- and inter-group genetic diversity based on pairwise nucleotide substitution differences.

	Within group distance (in percent)	
	Mean	Max
1a	1.02	2.8
1a1	0.63	1.6
1a2	0.60	1.5
1a3	0.13	0.2

	Between group distance (in percent)	
	Mean	Max
1a1 vs 1a2	1.39	2.5
1a1 vs 1a3	1.93	2.6
1a2 vs 1a3	2.09	2.8

The 1a1 and 1a2 subtypes had a similar mean genetic diversity of 0.63% and 0.60%, respectively. Two predicted amino acid differences were found between 1a1 and 1a2 subtypes in the most antigenic region VP1u, namely 30 V/L and 107 D/N, both in the Belarusian and in reference strains.

**Figure 7:**
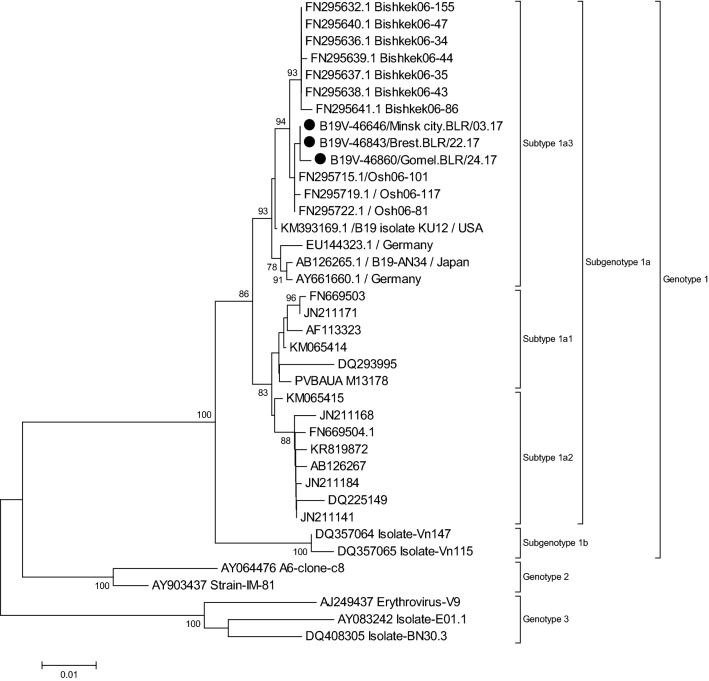
Phylogenetic tree based on 994 nt of the NS1/VP1-unique region junction. All sequences closely related to the 1a3 strains from Belarus available on Genbank were included. The sequences from Belarus are marked with a black dot. Only bootstrap values ≥ 70% (1000 replicates) are shown.

The mean between group genetic distance was 1.39% for subtypes 1a1 and 1a2, and reached 1.93% and 2.09% between each of these subtypes and 1a3, respectively (Table 2). Phylogenetic analysis of all genotype 1a sequences covering the 994 nt NS1/VP1u region downloaded from GenBank (November 2019) showed that only few sequences from Osh (n = 3), Bishkek (n = 7), US (n = 1), Japan (n = 1) and Germany (n = 2) grouped with the 1a3 strains from Belarus, forming a clearly distinct cluster of genotype 1a (Fig. 7). The within group mean distance for this cluster was 0.53%.

## Discussion

Diagnosis of B19V infection was introduced in Belarus as an additional investigation of cases that previously would have been classified only as “neither measles nor rubella”. Surveillance sensitivity for measles and rubella is considered adequate if at least two clinically compatible cases per 100,000 population per year are rejected after laboratory examinations^14^. In all but three years (2007, 2008, 2010), this indicator exceeded the minimal level (Table 1), demonstrating the reliable detection of patients with acute exanthema. Thus, even in the absence of mandatory notification of B19V infection, comprehensive data concerning epidemiological and molecular characteristics of EI in Belarus were obtained.

B19V was constantly an important cause of exanthematous diseases representing overall 27.8% of the measles/rubella negative cases (Table 1). Some studies have shown a lower proportion of B19V infection (e.g. 2.5% in patients from Brazil^5^ and 4.5% in patients from Ireland^15^), which is most likely due to patient selection, study location and period (high or low incidence settings and years) and/or assays used. In our study, only clinically suspected measles or rubella cases were investigated, which led to a high confirmation rate of EI, similar to what was reported from Finland (20%^4^), Bulgaria (22%^7^) and Brazil (31.8%^16^). Our EI incidence values, obtained by investigating only measles and rubella negative samples, certainly underestimate B19V incidence, because we did not consider other clinical presentations of B19V infection, such as isolated arthropathy or hepatitis, which are, however, considered to be rare^17,18^. A previous study investigating 942 people between less than 1 and 68 years of age from the general population in Belarus found an overall seroprevalence of 51.2%. Seropositivity increased from 9.8% in up to 4 year old children to 85.1% in 40–45 year olds, with the highest increase in the age group 5–9 years^19^. Thus, the seroprevalence data largely agree with our EI incidence results, which provide an approximation of B19V infection in Belarus, despite the high number of subclinical infections^20^ and our disregard of the rare clinical presentations of B19V infection.

The long-term dynamics confirmed a cyclic course of EI in Belarus with significant variation in annual incidence rates (0.17 versus 2.03). The highest rates were observed in 2006 and in 2015–2016, suggesting a quite long epidemic cycle similar to some other studies^21,22^. However, a slight increase in incidence was detected in between the major epidemics, potentially pointing to the more frequently reported 4–6 years periodicity^23,24^.

As is typical for moderate climate countries^15,23^, EI in Belarus has a winter-spring-early summer seasonality. Although the data clearly showed that EI is predominantly a childhood disease in Belarus, the third most frequently affected age group were 20–29 year old people, suggesting a risk of infection during pregnancy, with all the potential sequelae this may entail^24^. This is also confirmed by our analysis of non-immune hydrops fetalis: the mothers of the 12 cases identified in Belarus during 7 years of follow-up were 20–38 years of age, with a median of 28.5 years^25^.

The molecular investigation of B19Vs confirmed the predominance of subgenotype 1a. The only subgenotype 3b strain was detected in 2006 in a patient from Kazakhstan and has never been found in Belarus again, suggesting importation as also reported from the UK, Greece and Brazil^26–28^.

Although 66.2% of the sequenced strains belonged to 1a2, this subtype was not dominant during all years but was associated with both periods of increased incidence (Fig. 6). Co-circulation of two subtypes has been reported from the Netherlands and Brazil^12,27^, but there was no clear connection to outbreaks. In Japan, however, B19V strains belonging to a group named IV circulated during an epidemic in 1981–1982, while group II strains, probably imported from the US or Europe, caused the next epidemic in 1986–1987^29^. In Belarus, virus importation linked to an increased incidence was not detected, but interestingly, the prevalence of 1a1 was highest in the inter-epidemic period between 2009 and 2012. It cannot be excluded that the circulation of subtype 1a1 is quite stable and during the years of increased incidence (and high prevalence of subtype 1a2) is more difficult to detect than in low incidence years. There was no statistically significant difference concerning geographical regions, age of the patients, or seasonal distribution for the strains selected for sequencing during epidemic and inter-epidemic periods.

Like in other countries^12,13,27^, the average genetic distance between the subtypes 1a1 and 1a2 was small in Belarus (1.32%) with a similar within-group genetic distance of about 0.6%. These data and the lacking molecular information from before 2005, do not allow speculating that one of the subtypes is more ancient and/or has been circulating for a longer time-period in the country. At the same time, in the highly immunogenic N-terminus of VP1u there were two amino acid differences between the subtypes with one of them (30 V/L) located in the most significant neutralizing epitope. However, this change does not influence the structural conformation of this region and antibody binding^30^.

Analysis of three outlier strains detected in Belarus in 2017 within subgenotype 1a led to our suggestion that a third subtype, named in accordance with B19V nomenclature^31^ as subtype 1a3, exists. Although full genome sequencing will bring further clarity, the current data based on 994 bp of the NS1/VP1u region showed a high bootstrap support (93, Fig. 7) for the cluster containing the 1a3 sequences. The phylogenetic analysis done to determine subtypes 1a1 and 1a2 showed the same separation both for the full-genome sequences and for NS1 or VP1/2 sequences^12^. Current data suggest that 1a3 has only once been described as dominant, namely in Osaka in 1986–1987^32^. Since, it has occasionally been detected in different parts of Europe and the US and further research is required to determine its geographical spread and prevalence.

The data presented here demonstrate that high-quality surveillance of measles and rubella can also provide comprehensive data on non-notifiable rash and fever diseases such as EI. Our findings did not only highlight the prominent role of B19V in Belarus, but also contributed to a better understanding of EI epidemiology in the country and likely in general in temperate zones. The molecular analyses suggested a previously underestimated diversity of subgenotype 1a sequences on a global level with a potential third subtype 1a3.

## Materials and methods

### Ethics statement

The research is not a clinical trial and therefore does not need to be registered. No humans or animals were treated for the study. The study protocol was approved by the Medical council of the Ministry of Health, Republic of Belarus. The authors had no contact to the patients. The serum samples were collected by medical staff in hospitals or outpatient clinics in accordance with “Requirements for the organization and conducting of anti-epidemic measures aimed at preventing the introduction and spread of measles and rubella” (# 130, December 26, 2013) based on the Law of the Republic of Belarus “Sanitary and epidemiological well-being of the population” (January 7, 2012). Information regarding patient demographics was extracted from the medical records. Upon arrival at the National Measles and Rubella Reference Laboratory, a unique identifier number was attributed to each sample and was used for all data analyses. All methods were performed in accordance with the relevant guidelines and regulations.

### Specimens

In the frame of the nationwide measles/rubella surveillance in Belarus between 2005 and 2019, a total of 6318 serum samples from measles and rubella suspected cases between 0 and 70 years of age were collected. All samples found negative for both measles and rubella were tested for IgM antibodies against B19V with commercial ELISA kits (Biotrin (2005–2007), DRG (2008), Virion\Serion (2009–2017) or Euroimmun (2018–2019)). The kit used in a certain year depended on national procurement. All ELISA tests used had a sensitivity of at least 97.7% and a specificity of minimum 97.9% according to the kit manuals, suggesting that false-positive and false-negative results are very unlikely to occur. The assays were performed as recommended by the manufacturers and the results were interpreted qualitatively. For patients with multiple specimens, only the sample collected at the earliest time point was tested for B19V infection.

### EI incidence rate determination

Nationwide overall EI incidence rates were calculated for each year from 2005 to 2019 by dividing the aggregated number of laboratory-confirmed cases by the aggregated population or age group population using National statistics data (http://www.belstat.gov.by/).

The long-term trend was determined using regression analysis and was estimated by the average incidence growth rate. The monthly dynamics were estimated by the average long-term monthly data. To identify the seasonal rise, the Poisson method was used. The age distribution was studied in 9 age groups: up to 3 years old, 4–6, 7–10, 11–14, 15–19, 20–29, 30–39, 40–49, and ≥ 50 years old. High-risk age groups were identified by comparing incidence rate ratios (IRR). The group with the lowest incidence rate was used as reference. Confidence intervals (CI) were determined by the Student's method.

### DNA amplification and sequencing

Samples for genotyping were selected to represent all the different geographical regions of the country affected in a certain year. No other selection criteria such as age of the patients or sex were considered. DNA was extracted according to the manufacturer’s protocol from 200 µl of serum with the QIAamp DNA Blood Mini Kit (Qiagen). Viral DNA for sequencing was prepared by nested-PCR using previously described primers e1855f, e1863f, B19-R1 and B19-R2 for amplification of a 1,100-bp region spanning the NS1/VP1-unique region junction (NS1/VP1u)^28,33^. The PCR products were analysed in a 1.5% agarose gel stained with ethidium bromide. Products for sequencing were purified with the QIAquick PCR Purification Kit (Qiagen).

PCR products were sequenced with the BigDye Terminator v3.1 Cycle Sequencing Kit (Life Technologies), using the nested PCR primers on a capillary sequencer (models 3130/3500 Avant, Applied Biosystems). Sequences were edited using SeqScape Software v3.0 (Applied Biosystems) and then aligned with references in ClustalW (integrated in MEGA version 5).

### Phylogenetic analysis

Phylogenetic analysis using MEGA version 5^34^ was based on the Neighbor Joining and Kimura 2-parameter methods and the 994 nt NS1/VP1u proposed for genotyping^33^. Genetic distances based on the Kimura 2-parameter model were calculated with MEGA version 5. The best fitting model (T92 + G) was used to confirm topology.

The new sequences are available under GenBank accession numbers MN737618-MN737621, MN817160-MN817227, MT371078 and MT371079.
